# The molecular basis of the associations between non-alcoholic fatty liver disease and colorectal cancer

**DOI:** 10.3389/fgene.2022.1007337

**Published:** 2022-12-09

**Authors:** Ting Qiu, Weitao Hu, Zilan Rao, Taiyong Fang

**Affiliations:** ^1^ Department of Gastroenterology, The Second Affiliated Hospital of Fujian Medical University, Quanzhou, China; ^2^ Department of Rheumatology, The Second Affiliated Hospital of Fujian Medical University, Quanzhou, China

**Keywords:** non-alcoholic fatty liver disease (NAFLD), colorectal cancer (CRC), differentially expressed genes (DEGs), bioinformatics analysis, miRNA-gene regulatory network

## Abstract

**Background:** Given the ongoing research on non-alcoholic fatty liver disease (NAFLD) and colorectal cancer (CRC), the number of studies suggesting a strong link between NAFLD and CRC is on the rise, while its underlying pathological mechanisms remain uncertain. This study aims to explore the shared genes and mechanisms and to reveal the molecular basis of the association between CRC and NAFLD through bioinformatics approaches.

**Methods:** The Gene Expression Omnibus (GEO) dataset GSE89632 is downloaded for NAFLD cases and healthy controls. Additionally, the GSE4107 and GSE9348 datasets are obtained for CRC cases and healthy controls. Differentially expressed genes (DEGs) are obtained for NAFLD and CRC datasets, as well as shared genes between the two disorders. GO and KEGG enrichment analyses are further conducted. Subsequently, the STRING database and Cytoscape software are utilized to establish the PPI network and identify the hub genes. Then, co-expression analysis is performed using GeneMANIA. Subsequently, ROC curves and external datasets validation were applied to further screen the candidate markers. Finally, NetworkAnalyst is available as a means to construct a miRNA-gene regulatory network.

**Results:** Under the threshold of FDR ≤ 0.01, 147 common genes are obtained in NAFLD and CRC. Categorization of GO functions shows that DEGs are predominantly enriched in “response to organic substance”, “cellular response to chemical stimulus”, and “response to external stimulus”. The predominant KEGG pathways in DEGs are the “IL-17 signaling pathway”, the “TNF signaling pathway”, “Viral protein interaction with cytokine and cytokine receptor”, “Cytokine-cytokine receptor interaction”, and the “Toll-like receptor signaling pathway”. Additionally, *MYC*, *IL1B*, *FOS*, *CXCL8*, *PTGS2*, *MMP9*, *JUN*, and *IL6* are identified as hub genes by the evaluation of 7 algorithms. With the construction of miRNA-gene networks, 2 miRNAs, including miR-106a-5p, and miR-204-5p are predicted to be potential key miRNAs.

**Conclusion:** This study identifies possible hub genes acting in the co-morbidity of NAFLD and CRC and discovers the interaction of miRNAs and hub genes, providing a novel understanding of the molecular basis for the relevance of CRC and NAFLD, thus contributing to the development of new therapeutic strategies to combat NAFLD and CRC.

## Introduction

Non-alcoholic fatty liver disease (NAFLD), or more precisely, metabolism-related fatty liver disease, is a chronic and increasingly prevalent liver disease associated with insulin resistance (IR), obesity, hypertension, hyperlipidemia, and type 2 diabetes (T2DM) (Younossi, 2019). NAFLD, which affects one in four adults, is an umbrella term that encompasses a heterogeneous spectrum of diseases that initially evolves from hepatic steatosis leading to non-alcoholic steatohepatitis (NASH), cirrhosis, and ultimately hepatocellular carcinoma (HCC) (Younossi, 2019). NAFLD imposes substantial morbidity and mortality on those affected, with cardiovascular disease (CVD) being the most common contributor to death, followed by liver-related complications and extrahepatic malignancies (Angulo et al., 2015). As a clinicopathological syndrome that is challenging to diagnose and treat, NAFLD is a critical threat to global health and the economy.

Colorectal cancer (CRC) is a widespread malignancy that scares everyone. With the third highest incidence rate, CRC is the second leading cause of cancer-related deaths as per the Global Cancer Statistics 2020 (GLOBOCAN 2020) (Sung et al., 2021). CRC is not easily detectable in its early stages and, more frighteningly, once it progresses to advanced stages, the five-year survival rate is less than 10% (Parizadeh et al., 2019). Such high incidence, high mortality rate, and poor prognosis impose a heavy health and economic toll on families and society. This highlights the importance of identifying at-risk populations. Limiting the evolution of NAFLD may be a sensible method to prevent CRC.

Nowadays, the correlation between NAFLD and CRC has been confirmed by several studies. Risk factors for NAFLD, such as hyperlipidemia, obesity, and diabetes, have been also reported to be the predictable risk of CRC (Jimba et al., 2005). Individuals with NAFLD have a higher opportunity to suffer from CRC and a worse prognosis (Wu et al., 2019). Evidence from a meta-analysis suggests that both the existence and severity of NAFLD may be closely linked to an elevated risk of suffering from cancer (Chen et al., 2019). Similarly, a large cross-sectional study in Korea noted an elevated risk of CRC in patients with NAFLD who possessed higher non-invasive fibrosis scores (Ahn et al., 2017). To date, although a growing number of studies have verified the strong correlation between NAFLD and CRC, related pathogenetic and genetic studies are still limited. Further exploration of CRC in the setting of NAFLD, with potential therapeutic options, is needed.

Bioinformatics, as a discipline that can be applied to help people analyze the complex mechanisms of disease, has shown great ability in the study of a wide variety of diseases, including the identification of novel cancers (Yan et al., 2018; HA et al., 2020) and the prediction of novel biomarkers for diseases (Wang and Liotta, 2011; Tsai, 2019). Driven by the Gene Expression Omnibus (GEO) database, an accessible database offering genetic information that can be utilized for bioinformatic analysis to define new disease targets, a gene expression profile for NAFLD, and two gene expression profiles for CRC are downloaded. Differentially expressed genes (DEGs), as well as shared genes between NAFLD and CRC are ascertained following analysis. Clustering analysis *via* Gene Ontology (GO) terminology enrichment, Kyoto Gene and Genome Encyclopedia (KEGG) is performed to ascertain the functions and pathways of genes. Protein-protein interaction (PPI) network is applied to analyze the shared gene interactions and locate hub genes. Eventually, a predictive miRNA-gene regulatory network is constructed using NetworkAnalyst to predict key miRNAs associated with DEGs. This study aims to explore the pathological mechanisms and potential therapeutic targets of CRC in the context of NAFLD.

## Materials and methods

### Microarray data collection

**FIGURE 1:**
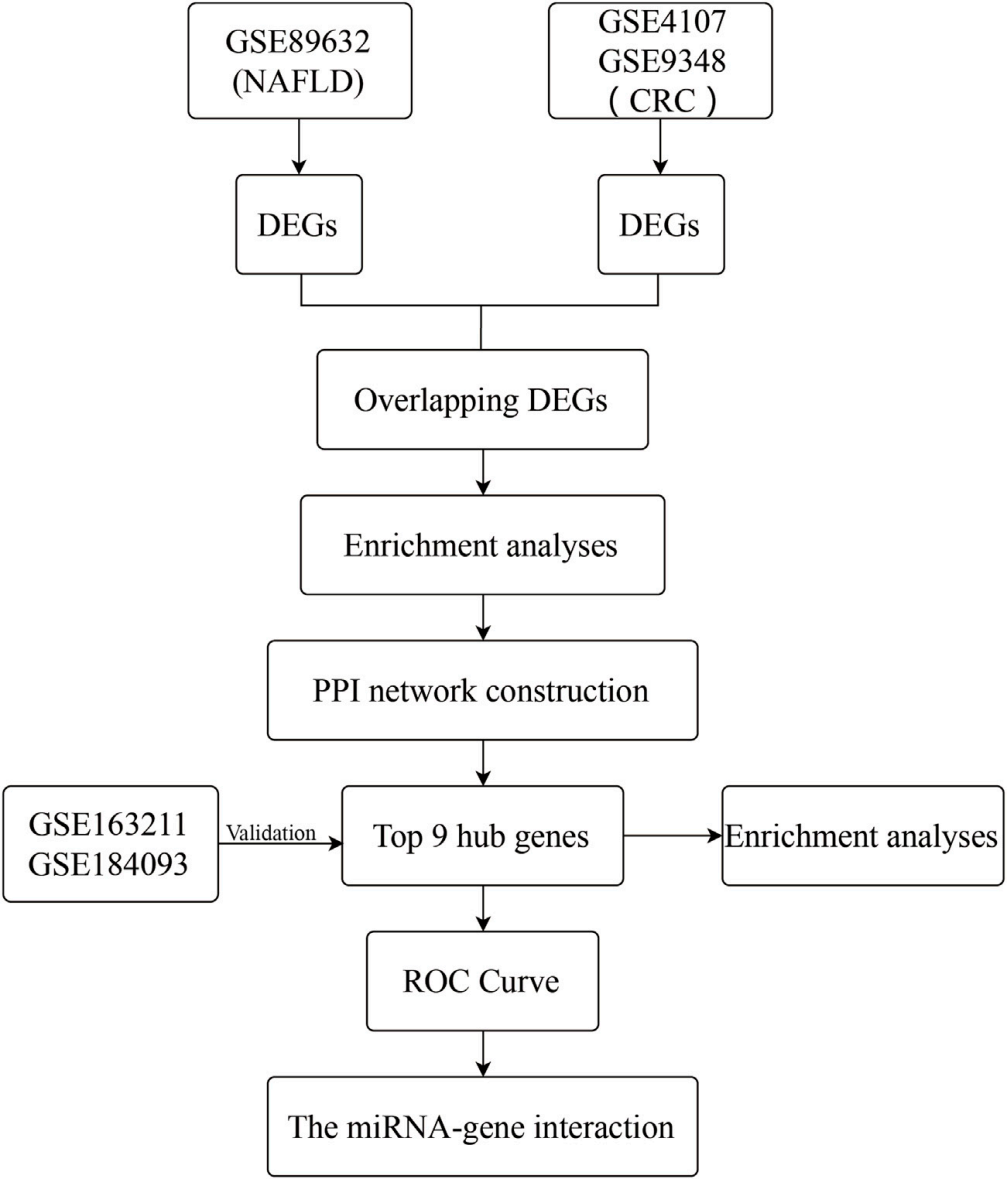
The specific flow map of this study.

From the Gene Expression Omnibus (GEO) database (http://www.ncbi.nlm.nih.gov/geo) (Edgar et al., 2002), microarray datasets for NAFLD and CRC are downloaded. The GSE89632 dataset (Arendt et al., 2015) contains RNA data from liver biopsies of patients with NAFLD (39 samples) and normal controls (24 samples). The GSE4107 dataset (Hong et al., 2007) contains colonic mucosal sample data from 12 CRC patients and 10 healthy controls. Information on colonic mucosal samples of 70 CRC patients and 12 non-CRC controls is in GSE9348 (Hong et al., 2010). Since these gene expression profiles are extracted from free and open databases available on the internet, Ethics Committee approval is not required for our study. The design block diagram of this study is shown in Figure 1.

### Differentially expressed genes selection

The two datasets GSE4107 and GSE9348 are normalized by log2-transformed [log2 (x + 1)] and removed from the batch effect for further analysis. The “limma” package from R software is used to identify the differentially expressed genes (DEGs) in the NAFLD and CRC datasets respectively. Probes without matching gene symbols and genes presenting multiple probes are excluded. For the purpose of reducing the false positive rate, the adjusted *p*-value (FDR) is taken. The selection criteria is set to FDR≤0.01 and |log2 FC|≥0.8. The screened DEGs for both diseases are put into the online analysis tool Venn (http://bioinformatics.psb.ugent.be/webtools/Venn/) to obtain intersecting genes. The shared genes are screened out for the next step of the analysis.

### Functional classification and pathway enrichment of differentially expressed genes

Gene ontology (GO) (Pomaznoy et al., 2018) and Kyoto Encyclopedia of Genes and Genomes (KEGG) (Kanehisa et al., 2017)Pathway analyses are performed using the analysis website Metascape (https://metascape.org/gp/index.html#/main/step1) (Zhou et al., 2019) to explore DEGs’ potential biological functions both in NAFLD and CRC. Values of *p* < 0.01 is assumed to be statistically significant.

### Establishment of protein-protein interaction and identification of hub genes

The PPI network is constructed through STRING (http://string-db.org/) (Franceschini et al., 2013) to further investigate the interactions between the shared genes which are obtained above. Interaction scores over 0.4 as significant are considered. Following this, it is visualized through the application of Cytoscape software to the PPI network (Franceschini et al., 2013). And then, the Cytoscape plugin, MCODE, is performed to filter out pivotal protein expression molecules. Set the selection criteria as: degree cut-off = 2, node score cut-off = 0.2, and max depth = 100. Subsequently, the cytoHubba plugin is applied for evaluation, and genes with at least 2 recurrences according to 7 algorithms (MCC, MNC, EPC, Degree, BottleNeck, Closeness, and Radiality) are taken as hub genes. Finally, GeneMANIA (http://www.genemania.org/) (Warde-Farley et al., 2010) is utilized to identify intra-gene associations and construct co-expression networks.

### Evaluation and verification of hub genes

The sensitivity and specificity of these hub genes in predicting NAFLD and CRC disease are assessed by the receiver operating characteristic (ROC) curve and the area under the ROC curve in NAFLD and CRC datasets. Additional publicly available datasets GSE163211 and GSE184093 are used to further validate the expression levels of these hub genes in both diseases. The two-sample *t*-test was used to compare the differential expression levels, and *p*-values <0.05 were regarded as statistically significant. The box plots were used to represent the expression levels of hub genes in different groups by the R package ggplot2. Statistical significance was indicated as follows: *(*p* < 0.05), **(*p* < 0.01), and ns (*p* > 0.05, no significance).

### MiRNAs associated with hub genes

A miRNA-gene regulatory network of hub genes is to be built with the NetworkAnalyst tool (version 3.0, https://www.networkanalyst.ca/), followed by the application of Cytoscape software for collation and visualization.

## Results

### Differential expression analysis of differentially expressed genes in non-alcoholic fatty liver disease and colorectal cancer

**FIGURE 2:**
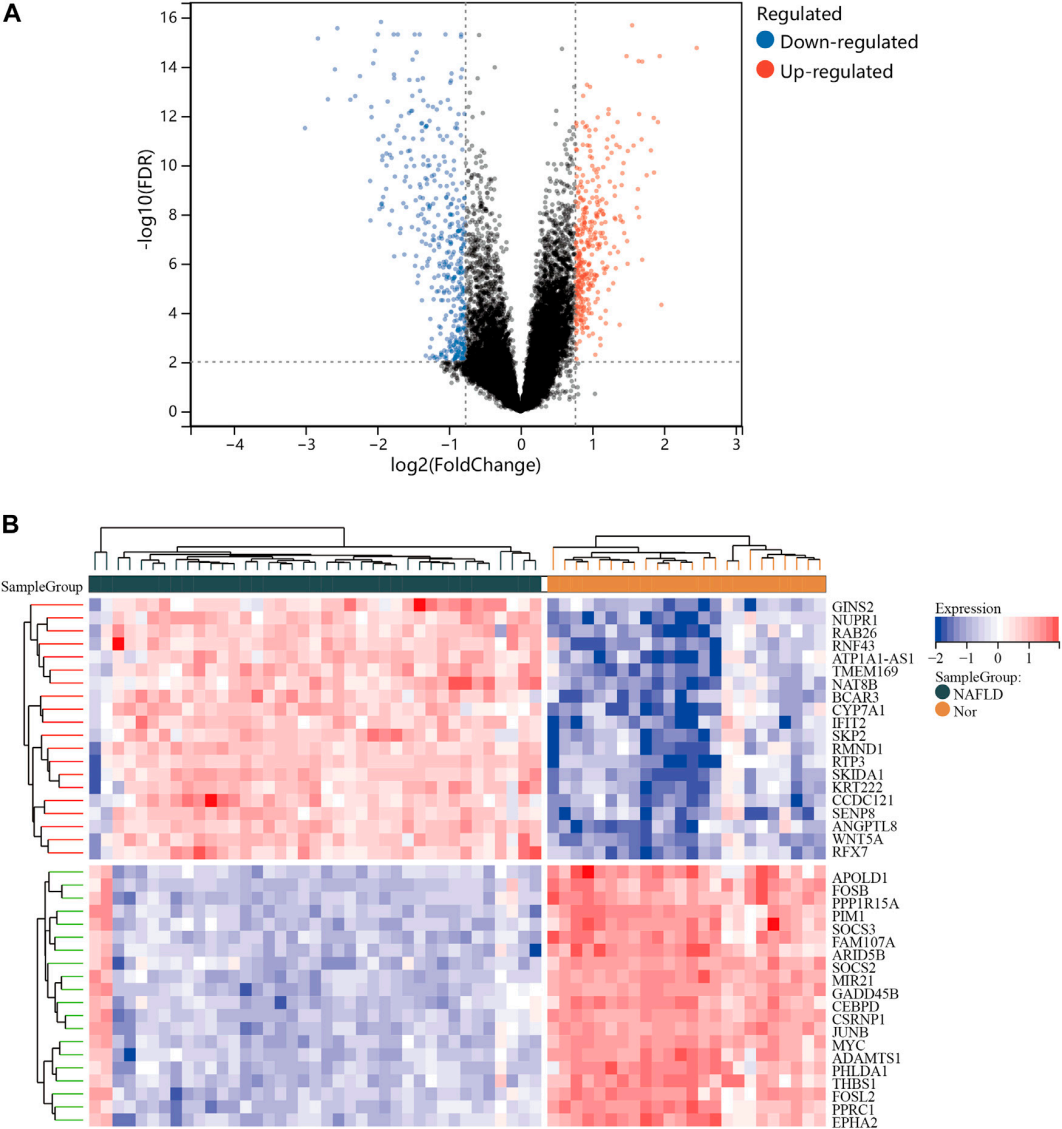
Differentially expressed genes (DEGs) for the GSE89632 dataset. **(A)**, volcano plot depicting the status of gene expression between patients with NAFLD and normal controls. **(B)**, heatmap of the top 40 DEGs. Regions in red and blue are displayed to distinguish between the up- and down-regulation of genes.

**FIGURE 3:**
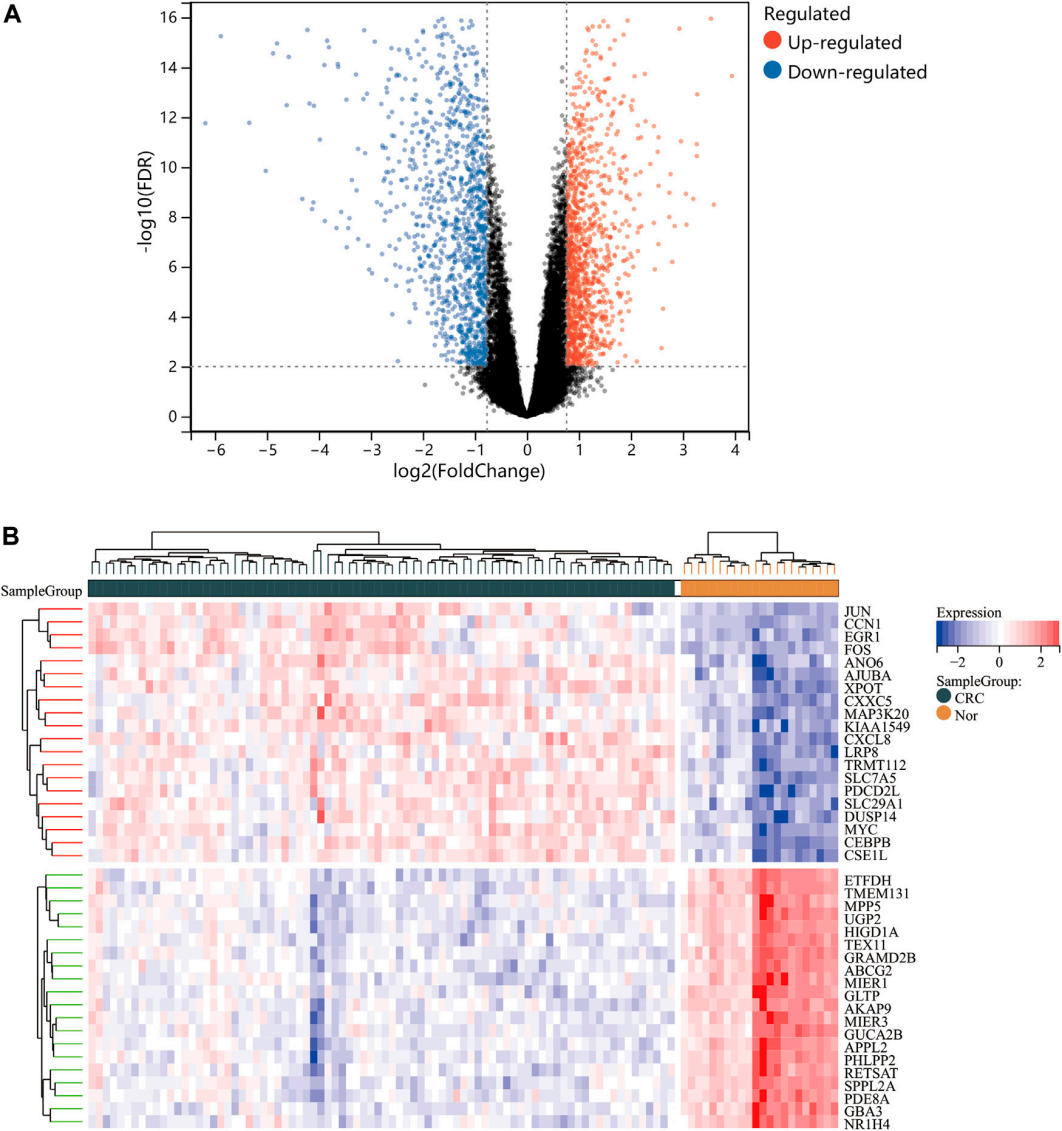
Differentially expressed genes (DEGs) for the dataset derived by integrating GSE4107 and GSE9348. Panel **(A)**, volcano plot depicting the status of gene expression between patients with CRC and normal controls. Panel **(B)**, heatmap of the top 40 DEGs. Regions in red and blue are displayed to distinguish between the up- and down-regulation of genes.

**FIGURE 4:**
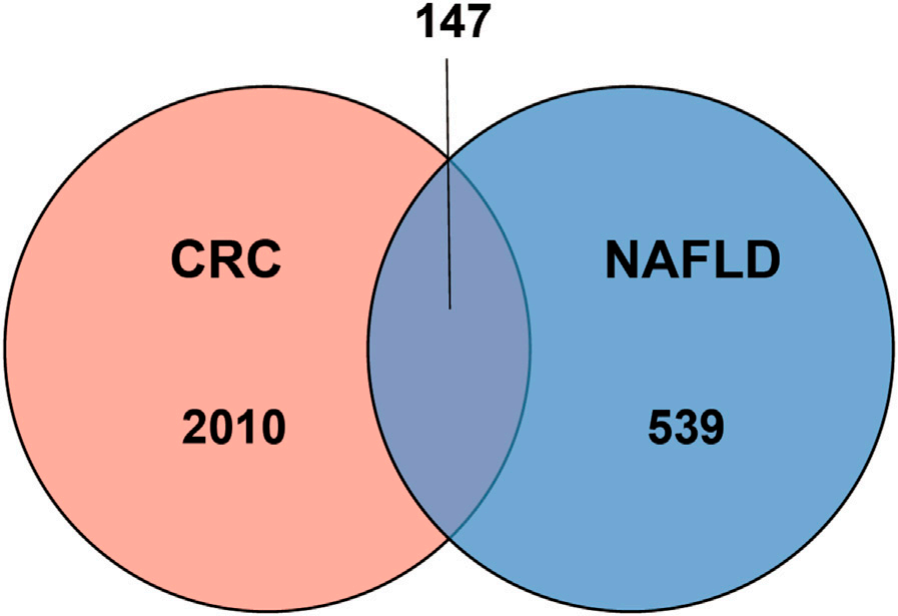
Venn diagram depicting shared genes between NAFLD and CRC.

One dataset containing NAFLD gene information (GSE89632) and two datasets containing CRC gene information (GSE4107, GSE9348) are retrieved from the NCBI GEO database. Details about the three datasets are shown in Table 1. After preprocessing, we obtained 686 DEGs in NAFLD group and 2,157 DEGs in CRC samples. The DEGs are visualized with volcanos (Figures 2A, 3A) and heat maps (Figures 2B, 3B). Further screening of these genes is performed and Venn diagrams are plotted. 147 intersecting genes common to both diseases are obtained, as shown in Figure 4.

**TABLE 1:** Information on the datasets included in the current study.

**Dataset**	**Reference**	**Platform**	**Source**	**No. of samples (Controls/Cases)**
GSE89632	Arendt et al. (2015)	GPL14951	liver	24/39
GSE4107	Hong et al. (2007)	GPL570	colonic mucosa	10/12
GSE9348	Hong et al. (2010)	GPL570	colonic mucosa	12/70

### GO and KEGG enrichment pathway analysis of overlapping differentially expressed genes

**FIGURE 5:**
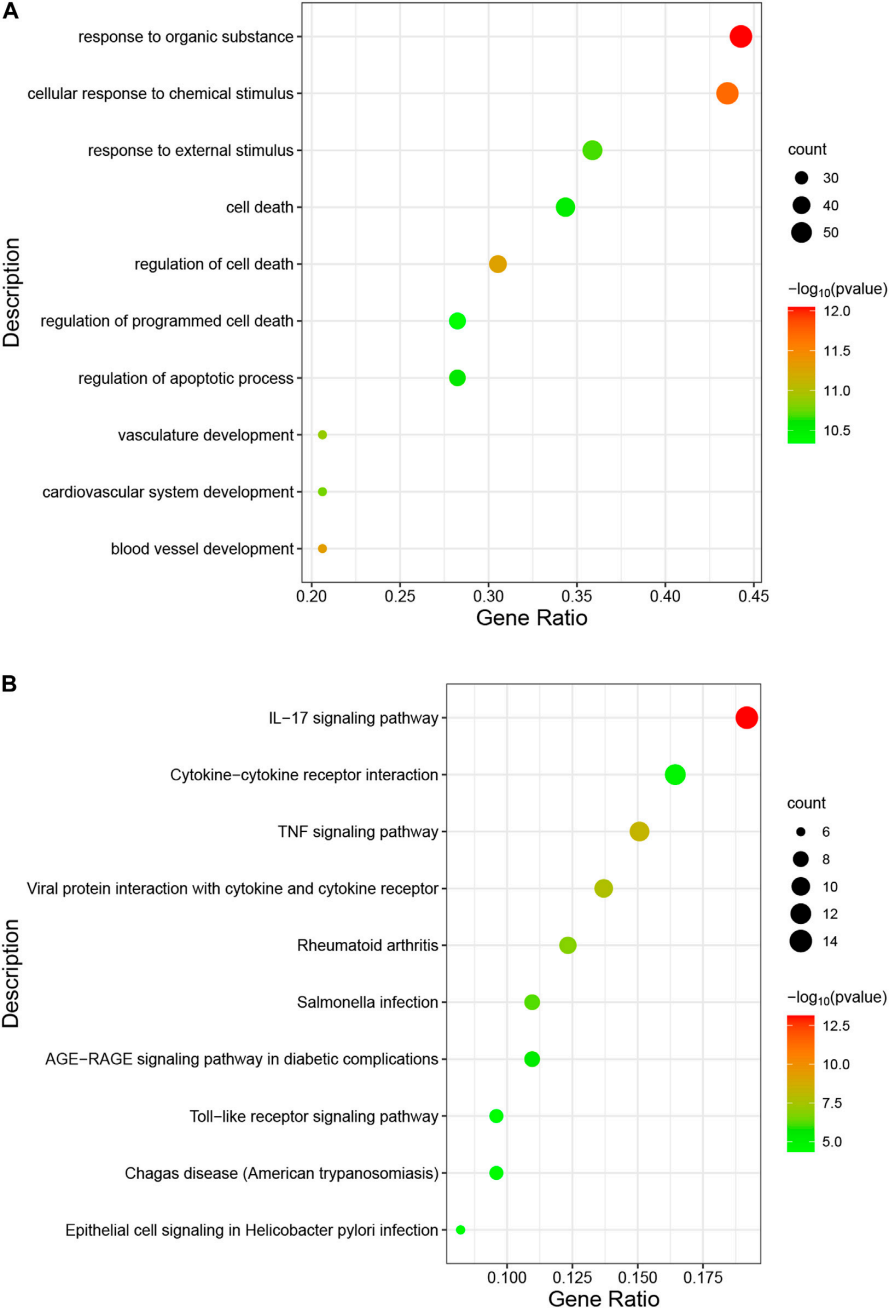
Analysis of functional enrichment for intersecting common genes. **(A)**, bubble diagram of GO analyses for common genes. **(B)**, bubble diagram of KEGG analyses for common genes. The number of genes is indicated by the size of the circle, whereas the *p*-value is shown in gradient color.

The occurrence of diseases usually relates to multiple biological functions. GO analysis shows that the overlapping genes are mainly enriched in “response to organic substance”, “cellular response to chemical stimulus”, and “response to external stimulus” (Figure 5A). The predominant KEGG pathways in DEGs are the “IL-17 signaling pathway”, the “TNF signaling pathway”, “Viral protein interaction with cytokine and cytokine receptor”, “Cytokine-cytokine receptor interaction”, and the “Toll-like receptor signaling pathway” (Figure 5B). The results indicate an important role of inflammation in these two diseases.

### Establishment of protein-protein interaction and identification of hub genes

**FIGURE 6:**
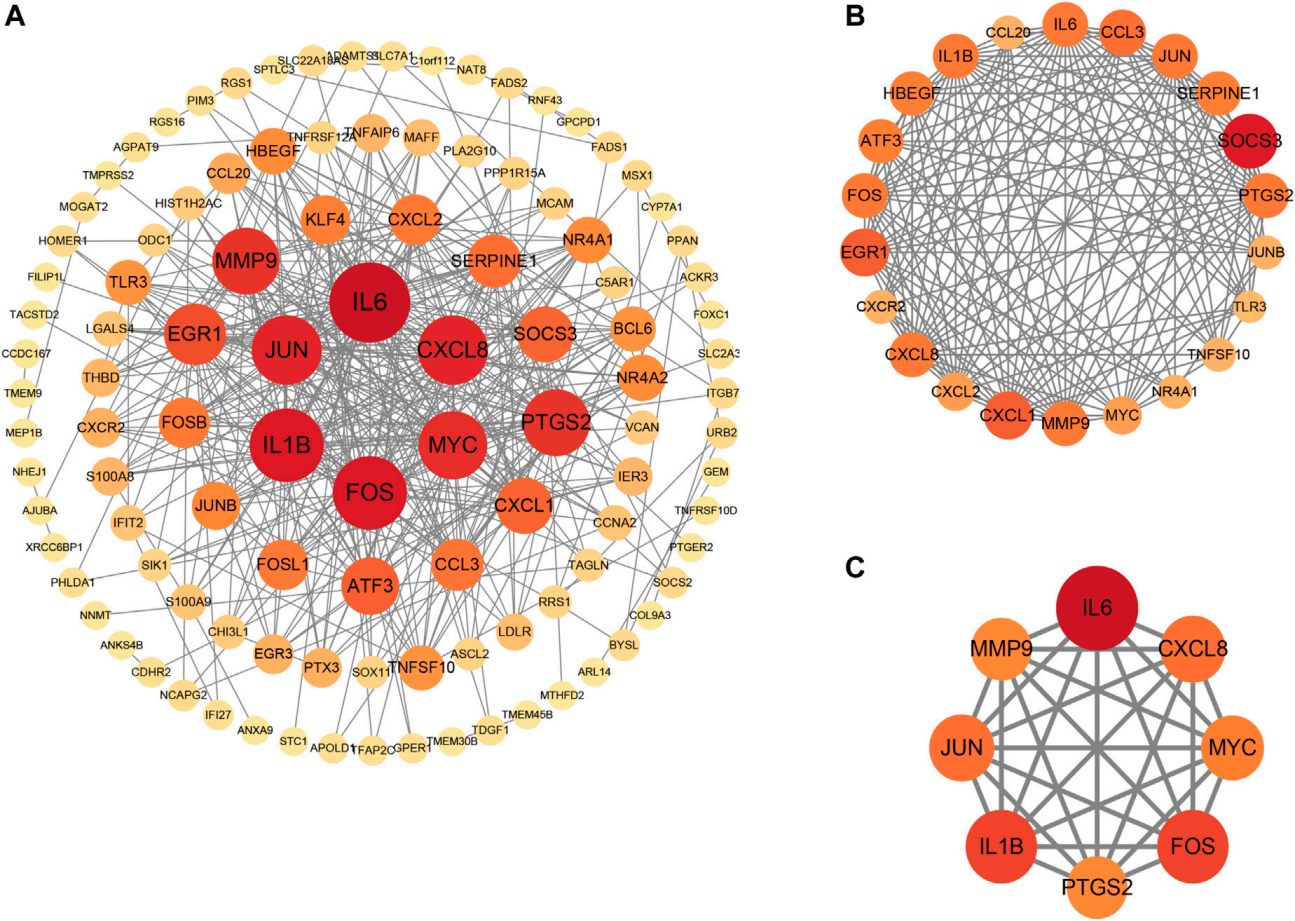
Protein-protein interaction (PPI) networks. **(A)**, PPI of 147 shared genes between NAFLD and CRC identified. **(B)**, the regions most densely connected as identified by the MCODE plugin. **(C)**, 8 hub genes were ascertained with 7 different algorithms of cytoHubba. Corresponding to the nodes, the size and color of each gene is based on the degree of interaction.

**FIGURE 7:**
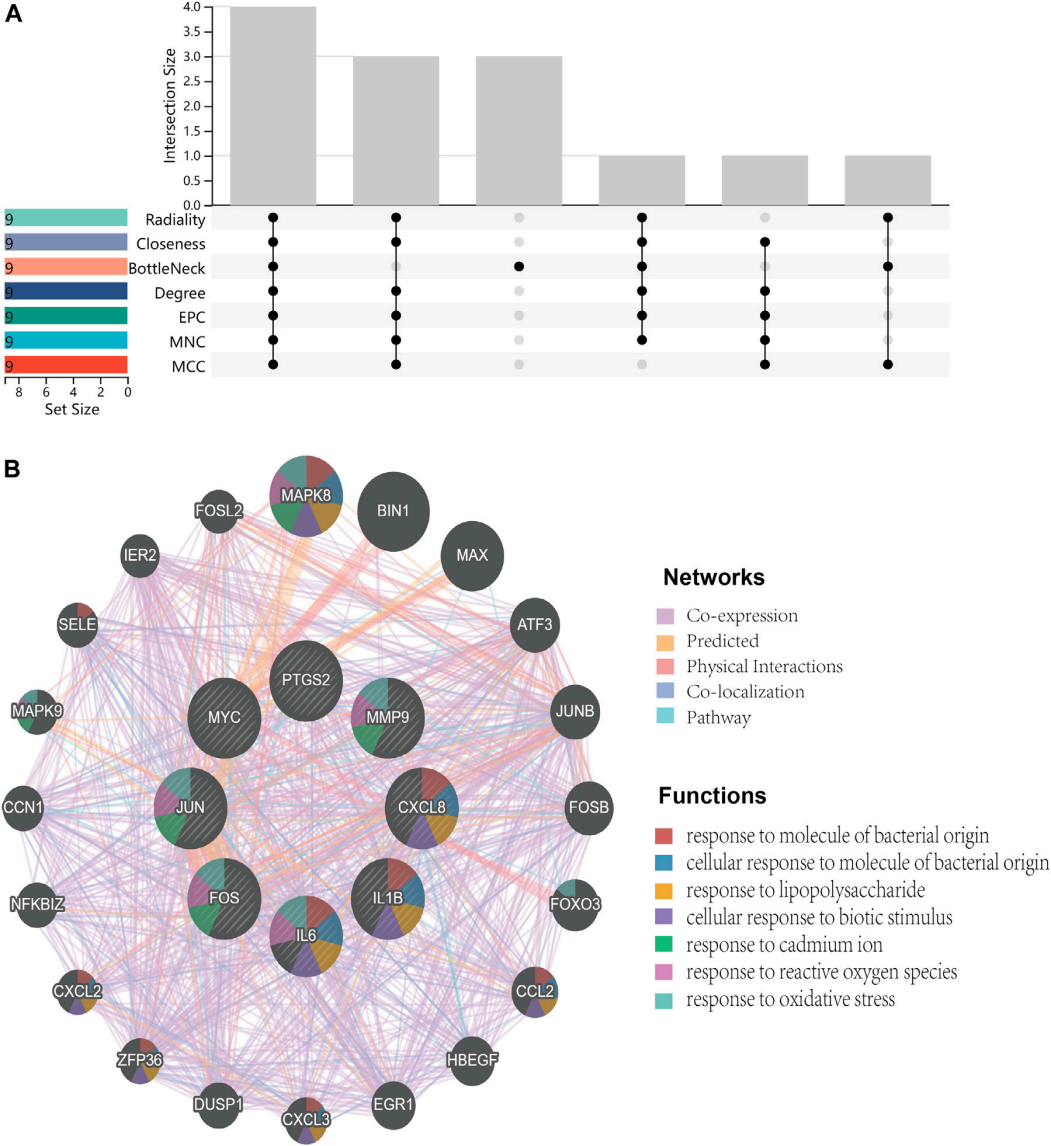
Venn diagram and co-expression network. **(A)**, Venn diagram illustrates the situation of 7 algorithms to identify hub genes. Each algorithm selects the top 9 ranked genes. **(B)**, Co-expression networks of hub genes are constructed by GeneMANIA. The networks are represented by different color blocks inside the circles and the functions are represented by various line colors.

**FIGURE 8:**
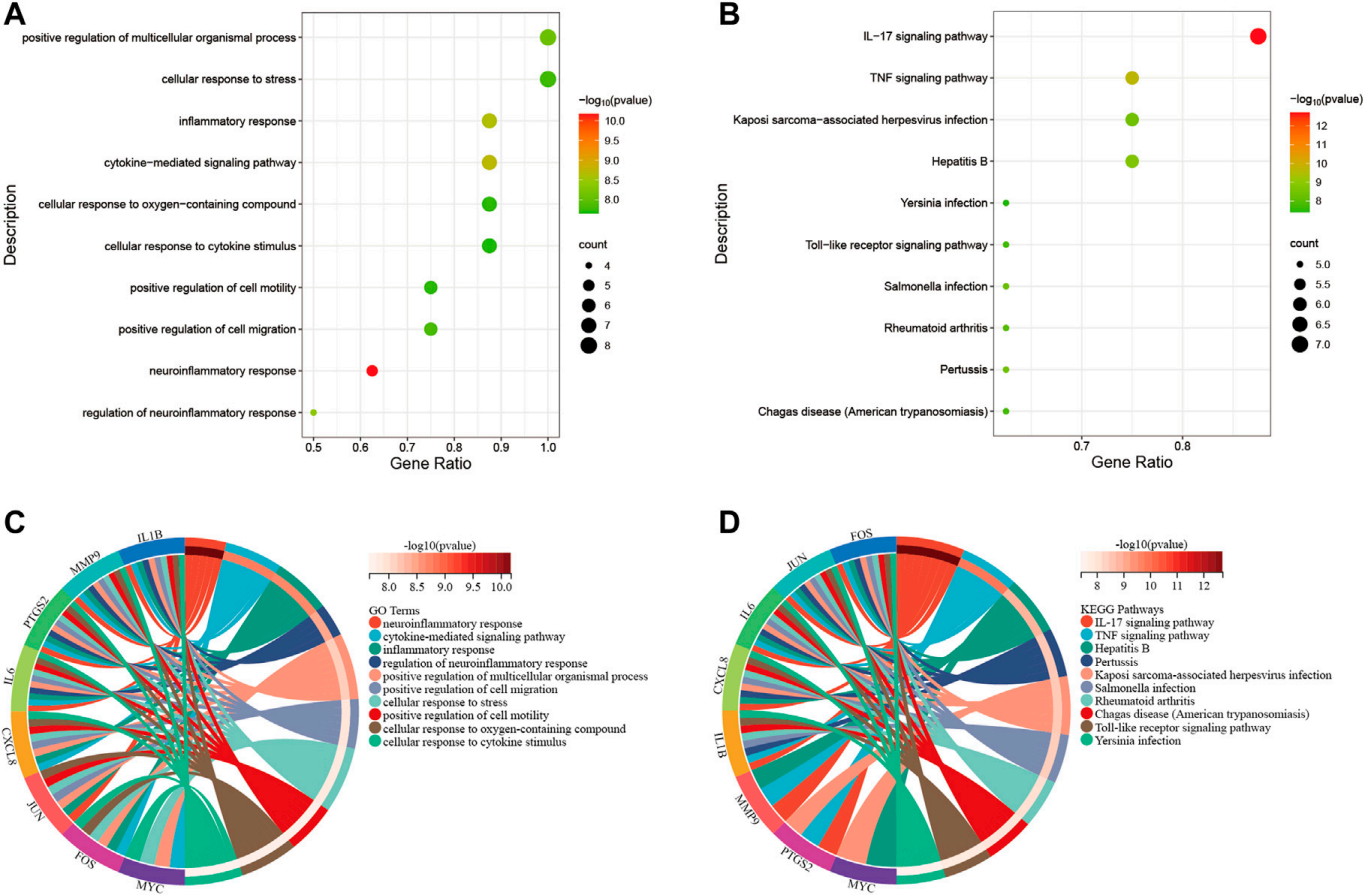
Functional enrichment analysis. **(A)**, bubble diagram that shows GO analysis of 8 hub genes. **(B)**, bubble diagram that shows the KEGG analysis of 8 hub genes. **(C)**, circle diagram to display the GO terms exactly enriched for each of the 8 hub genes. **(D)**, circle diagram to display the KEGG pathways exactly enriched for each of the 8 hub genes. Distinguish different enrichment terms with various colors.

Primarily, PPI networks are conducted to determine the interactions among DEGs *via* the online Search Tool for the Retrieval of Interacting Genes (STRING). Then, visual analysis is performed using Cytoscape (Figure 6A). Based on Cytoscape’s plugin MCODE, The most tightly attached region (22 nodes, 161 edges) of the PPI network is identified (Figure 6B). Table 2 shows the ranking of the top 9 hub genes in the network according to 7 algorithms of cytoHubba. After taking the intersection of the Venn diagrams, the top 8 genes are determined as potential hub genes, including *IL6* (Interleukin-6), *JUN* (Transcription factor AP-1), *PTGS2* (Prostaglandin G/H synthase 2), *MMP9* (Matrix metalloproteinase-9), *CXCL8* (Interleukin-8), *FOS* (Proto-oncogene c-Fos), *IL1B* (Interleukin-1 beta), and *MYC* (Myc proto-oncogene protein) (Figures 6C, 7A). GO analysis indicates that the 8 hub genes are mainly enriched in “neuroinflammatory response”, “cytokine-mediated signaling pathway”, “inflammatory response”, “regulation of neuroinflammatory response”, “positive regulation of cell migration”, and “cellular response to cytokine stimulus” (Figure 8A). In the KEGG analysis, the mainly enriched pathways are the “IL-17 signaling pathway”, the “TNF signaling pathway”, “Kaposi sarcoma-associated herpesvirus infection”, “Hepatitis B”, and the “Toll-like receptor signaling pathway” (Figure 8B). Majority of hub genes are involved in the IL-17 and TNF signaling pathways, as well as inflammatory responses. The details of each hub gene and its enrichment pathways are depicted in Figures 8C,D.

**TABLE 2:** List of the top 9 genes ranked by 7 algorithms in cytoHubba.

MCC	MNC	EPC	Degree	BottleNeck	Closeness	Radiality
*IL6*	*IL6*	*IL1B*	*IL6*	*IL6*	*IL6*	*IL6*
*JUN*	*FOS*	*IL6*	*FOS*	*MYC*	*IL1B*	*IL1B*
*PTGS2*	*IL1B*	*FOS*	*IL1B*	*CXCL1*	*FOS*	*FOS*
*CXCL8*	*CXCL8*	*JUN*	*CXCL8*	*MMP9*	*CXCL8*	*CXCL8*
*FOS*	*JUN*	*CXCL8*	*JUN*	*JUN*	*JUN*	*MMP9*
*IL1B*	*MMP9*	*PTGS2*	*MYC*	*SERPINE1*	*MMP9*	*PTGS2*
*MMP9*	*PTGS2*	*MMP9*	*MMP9*	*PTGS2*	*MYC*	*JUN*
*EGR1*	*MYC*	*EGR1*	*PTGS2*	*IER3*	*PTGS2*	*MYC*
*CXCL1*	*EGR1*	*MYC*	*EGR1*	*TNFAIP6*	*EGR1*	*CXCL1*

The italic values provided in Table represent the names of the genes.

### Evaluation and verification of hub genes

**FIGURE 9:**
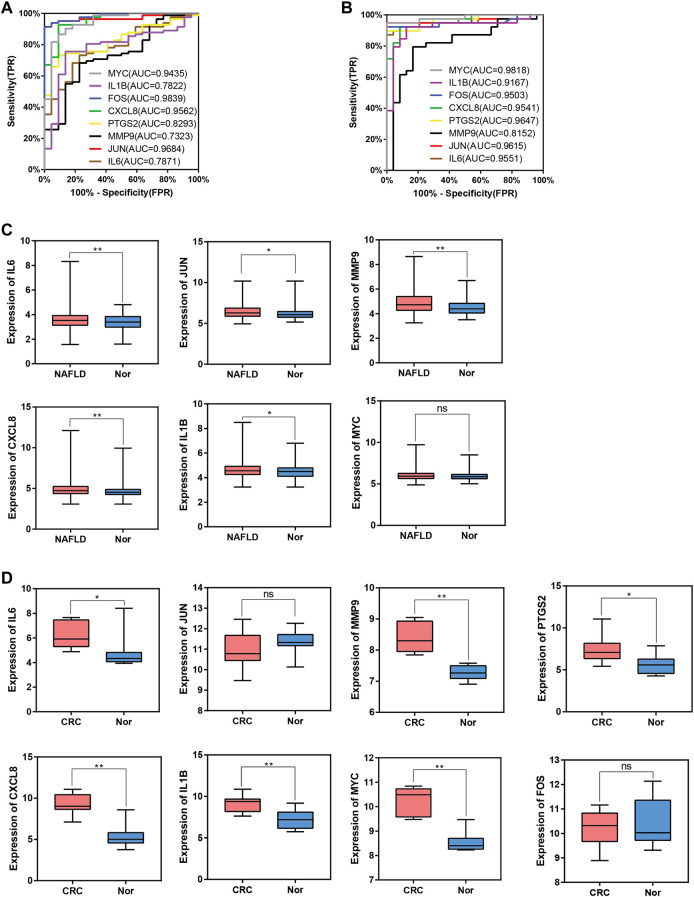
ROC curve and box plot of hub genes. **(A)**, The AUC of hub genes for the diagnosis of NAFLD in the ROC analysis of the GSE89632 dataset. **(B)**, The AUC of hub genes for the diagnosis of CRC in the ROC analysis of the dataset derived by integrating GSE4107 and GSE9348. **(C)**, The expression of hub genes in NAFLD vs. Normal group in the GSE163211 dataset. **(D)**, The expression of hub genes in CRC vs. Normal group in the GSE184093 dataset.

To evaluate whether the 8 hub genes are of predictive significance, ROC curve analysis is performed on the NAFLD and CRC datasets. It is shown that the AUCs of all hub genes are greater than 0.7 (Figures 9A,B). Microarray datasets retrieved from the GEO database for NAFLD and CRC (GSE163211: 242 NAFLD samples vs. 76 healthy controls; GSE184093: 9 CRC samples vs. 9 healthy controls) are used to validate the expression levels of hub genes after normalization. In GSE163211, *FOS* and *PTGS2* are not involved in the quantification of expression from NAFLD patients. The expression profiles of the other 6 hub genes in GSE163211 are shown in Figure 9C. Compared with the normal group, *IL6*, *MMP9*, *CXCL8*, and *IL1B* in GSE163211 are significantly increased. The expression profiles of 8 hub genes in CRC patients are validated by the GSE184093 dataset. It is observed that the expression levels of *IL6*, *MMP9*, *PTGS2*, *CXCL8*, *IL1B*, and *MYC* are significantly higher than those in the normal group (Figure 9D). Therefore, combining the expression levels of hub genes and ROC results, we conclude that *IL6*, *CXCL8*, *MMP9* and *IL1B* may be the most potential candidate markers.

### The miRNA-gene regulatory network

**FIGURE 10:**
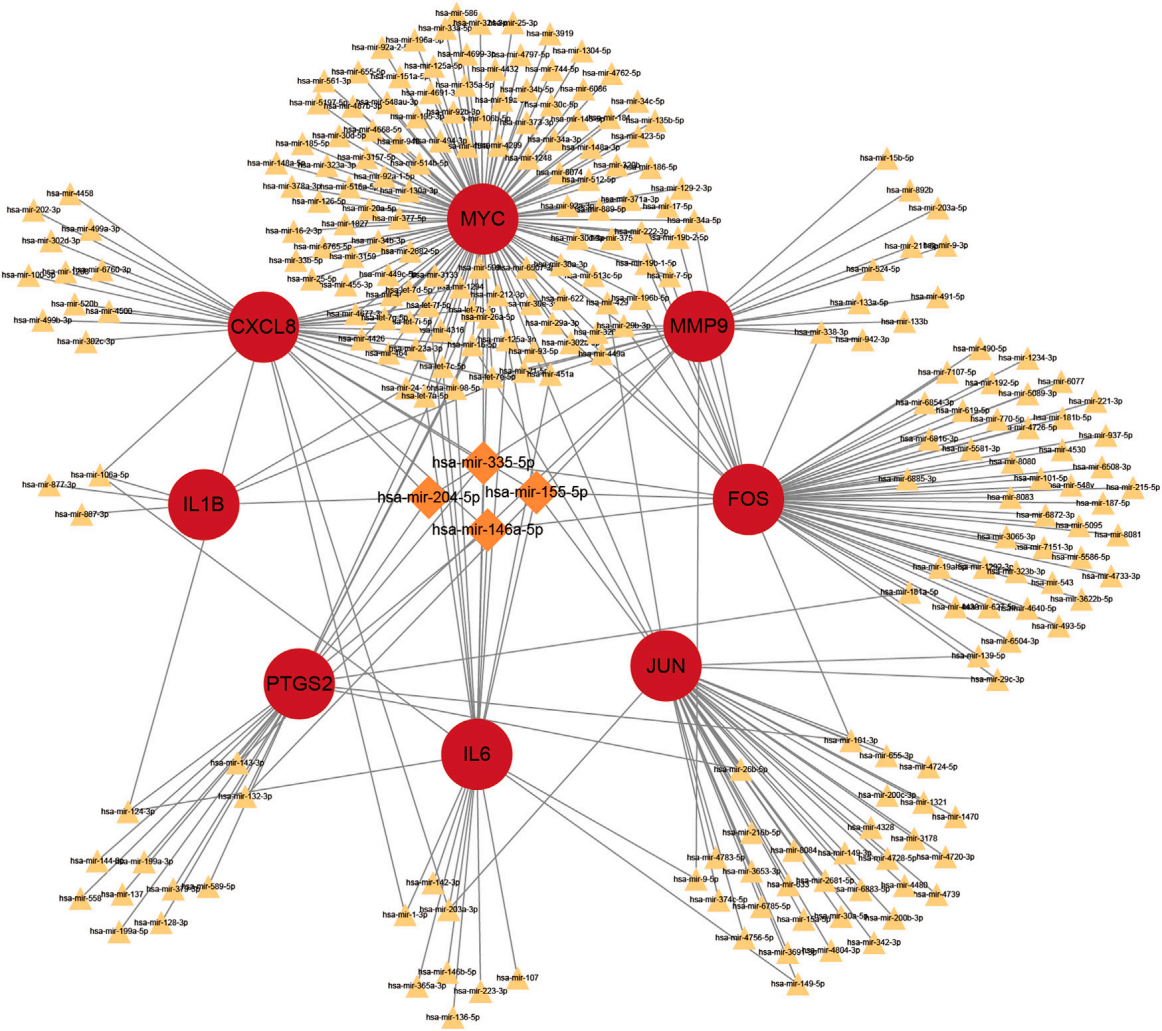
The top 4 hub genes in the integrated miRNA-gene network. The red circles indicate these hub genes. MiRNAs with linkage to more central genes are highlighted exclusively with larger orange diamonds, and the rest are indicated with smaller yellowish triangles.

Prediction of target miRNAs for hub genes is performed *via* the NetworkAnalyst database. As shown in Figure 10, in the miRNA-gene regulatory network, miR-106a-5p interacts with *IL6*, *CXCL8* and *IL1B*. In addition, miR-204-5p is a target miRNA for *MMP9*, *CXCL8* and *IL1B*.

## Discussion

NAFLD has been characterized as a chronic and ongoing pathological process evolving from hepatic steatosis to marked liver damage, affecting not only the liver itself but also being a promoter of carcinogenesis in other distant organs (Yang et al., 2017). A cohort study involving 1314 CRC patients who underwent surgical resection first confirmed that NAFLD is probably an independent factor impacting the overall survival (OS) of CRC victims (You et al., 2015). With an increasingly widespread impact, NAFLD has also emerged as a risk factor for CRC, which carries the second-highest global cancer mortality rate. Therefore, it would be worthwhile to address the underlying molecular mechanisms between NAFLD and CRC as well as to identify early targets for preventing disease progression.

In this study, the GEO dataset for NAFLD and CRC is searched and 147 common DEGs are identified. Categorization of GO functions reveals that DEGs are mainly enriched in “response to organic substance”, “cellular response to chemical stimulus”, and “response to external stimulus”. DEGs are mainly enriched in the “IL-17 signaling pathway”, the “TNF signaling pathway”, “Viral protein interaction with cytokine and cytokine receptor”, “Cytokine-cytokine receptor interaction”, and the “Toll-like receptor signaling pathway” concerning the KEGG pathway. The 8 hub genes identified with the application of 7 algorithms include *MYC*, *IL1B*, *FOS*, *CXCL8*, *PTGS2*, *MMP9*, *JUN*, and *IL6*. GO analysis indicates that the 8 hub genes are mainly enriched in “neuroinflammatory response”, “cytokine-mediated signaling pathway”, “inflammatory response”, “regulation of neuroinflammatory response”, “positive regulation of cell migration”, and “cellular response to cytokine stimulus”. In the KEGG analysis, the mainly enriched pathways are the “IL-17 signaling pathway”, the “TNF signaling pathway”, “Kaposi sarcoma-associated herpesvirus infection”, “Hepatitis B″, and the “Toll-like receptor signaling pathway”. The above analysis reveals that inflammatory processes may play an important role in the co-pathogenesis of NAFLD and CRC. Combining the expression of hub genes in validation datasets and ROC curves, IL6, MMP9, CXCL8, and IL1B are further identified as potential genes for the development of CRC based on NAFLD. Persistent lipid deposition in NAFLD induces a sustained inflammatory response with infiltration of lymphocytes (Chakraborty and Wang, 2020), macrophages, and neutrophils in the liver, which may provide a beneficial microenvironment for the initiation and further development of the metastatic CRC. Lymphocytes and macrophages that infiltrate may secrete pro-inflammatory cytokines like TNF-α(25), which evokes epithelial-to-mesenchymal transition (EMT) (Bates and Mercurio, 2003) and contributes to the metastasis of CRC. Infiltrating neutrophils secrete IL-17, which increases the inflammatory response in the hepatic ductal tract (Gao and Tsukamoto. -, 2016). Interleukin 17 (IL-17) is considered to be a pro-inflammatory cytokine and has been demonstrated to promote the transition from simple steatosis to steatohepatitis (Tang et al., 2011). IL-17 is able to promote tumor angiogenesis (Numasaki et al., 2003) by inducing the expression of vascular endothelial growth factor (VEGF), which acts as a pathogenic role in CRC formation (Li et al., 2019a). Hepatic TLR signaling is activated in NAFLD, and TLR2, TLR4, TLR5, TLR9 are all implicated in the pathogenesis of NAFLD. Therefore, Kouichi Miura et al. suggested that TLRs are targets for NAFLD treatment (Miura and Ohnishi, 2014). Toll like receptor signaling pathway can influence CRC progression through multiple pathways and is critical in the treatment of CRC (Moradi-Marjaneh et al., 2018). These enrichment results for GO terms and the KEGG pathway indicate that hub genes found in our study might be participating in the disease progression of NAFLD and CRC by the aforementioned means.

MMP9 is one of the zinc-ion-dependent metalloproteinase Family. It was revealed that overexpression of MMP9 could accelerate CRC progression and metastasis through the MKK-3/p38/NF-κB pathway (Chen et al., 2020). A cross-sectional study found that lower MMP9 levels and higher IL6 levels were associated with worse fibrosis by measuring 12 serum markers in 105 adult patients with NAFLD (Goyale et al., 2021). Overexpression of IL6 favors the progression of liver inflammation and NAFLD (Fontes-Cal et al., 2021). IL-6 contributes to the progression of CRC *via* the activation of STAT3 and NF-kB (De Simone et al., 2015). CXCL8, also known as IL-8, is necessary for the linkage between tumors and inflammation. CXCL8 plays an essential role in promoting angiogenesis, proliferation, invasion, migration and epithelial-mesenchymal transition (EMT) of CRC cells (Bie et al., 2019). The serum levels of CXCL8 were significantly higher in NASH patients compared to healthy group (Bahcecioglu et al., 2005). It was confirmed in a study involving 604 patients with NAFLD that common variants of IL6 and IL1B may increase susceptibility for NASH (Nelson et al., 2016). Higher expression of IL1B was detected in the visceral adipose tissue of CRC patients than in healthy controls (Castellano-Castillo et al., 2018). *IL6*, *MMP9*, *CXCL8* and *IL1B* are all involved in the IL17 signaling pathway and are inextricably linked to the inflammatory response. It is speculated in this study that *IL6*, *MMP9*, *CXCL8* and *IL1B* may contribute to the occurrence and development of CRC and NAFLD, and may be potentially candidate markers. Inflammation may be the access pathway that connects the co-occurrence of NAFLD and cancer.

The miRNA-gene regulatory network is constructed in this study, then the top 2 miRNAs interacting with DEGs are selected (miR-106a-5p, and miR-204-5p). The aberrant miRNA expression is tightly associated with cancer development and progression. MiR-204-5p exerts a potent tumor suppressor function in colorectal cancer by repressing RAB22A (a member of the RAS oncogene family) (Yin et al., 2014). Whether miR-204-5p affects NAFLD has not been studied. Research confirms that miR-204-5p may inhibit inflammation and chemokine production by regulating the IL6/IL6R axis (Li et al., 2019b). The possibility that miR-204-5p can act on NAFLD through the inhibition of inflammatory factors still needs further exploration. MiR-106a-5p can exert an influence on NAFLD progression through the NF-κB signaling pathway (Li et al., 2021). It was found that miR-106a-5p expression was elevated in CRC tissues, and patients with high miR-106a-5p expression tended to have a poor prognosis (Yue et al., 2015). The miR-204-5p obtained in the miRNA-gene regulatory network inhibited CRC and inflammation, while the overexpression of miR-106a-5p worsened CRC and NAFLD. Therefore, it was suggested that miR-204-5p and miR-106a-5p might be used as targets against NAFLD and CRC. NAFLD is often accompanied by inflammatory environment, and overexpression of inflammatory and chemokines has a pro-tumor role in CRC development. The 4 candidate markers screened in this study were all enriched in inflammatory responses and inflammation-related signaling pathways such as IL17 signaling pathway. Elevated IL6, MMP9, CXCL8 and IL1B in NAFLD may act on the IL17 signaling pathway and miR-106a-5p to promote the development and progression of CRC.

In conclusion, the current study identifies 8 hub genes by bioinformatics analysis and constructs a miRNA-gene regulatory network that helps to understand the pathophysiological processes of CRC and NAFLD as well as provides the potential early diagnostic and therapeutic targets for CRC and NAFLD. Nevertheless, there are several limitations to our study. One limitation of the study is that only the possible hub genes and miRNAs that act together in both NAFLD and CRC diseases are analyzed by bioinformatics, but the specific mechanisms are still lacking and need to be further explored. In addition, the finding is only based on predictions derived from public databases. Therefore, further biological and clinical data are needed to clarify the mechanism of interaction between these hub genes and miRNAs between CRC and NAFLD diseases.

## Conclusion

This study identifies a potential miRNA-gene regulatory network that may be relevant to CRC and NAFLD. 8 hub genes (including *MYC*, *IL1B*, *FOS*, *CXCL8*, *PTGS2*, *MMP9*, *JUN*, and *IL6*) are likely to exert an essential impact on the pathophysiological mechanisms of the two diseases. *IL6*, *MMP9*, *CXCL8* and *IL1B* were further identified as candidate markers. Several possible target miRNAs (miR-106a-5p, and miR-204-5p) are also predicted. This study provides new insights into the occurrence of NAFLD and CRC at the molecular level, which may be helpful in the early prevention of CRC and the development of treatment strategies for NAFLD and CRC. It also reminds us that patients with NAFLD or those with corresponding risk factors should be screened or alerted to the occurrence of CRC.

## Data Availability

The original contributions presented in the study are included in the article/Supplementary Material, further inquiries can be directed to the corresponding author.
